# Preparation, Characterization and Study of the Dissociation of Naproxen from Its Chitosan Salt

**DOI:** 10.3390/molecules27185801

**Published:** 2022-09-07

**Authors:** Ricardo S. Medeiros, Ana P. G. Ferreira, Tiago Venâncio, Éder T. G. Cavalheiro

**Affiliations:** 1 Instituto de Química de São Carlos, Universidade de São Paulo, Av. Trabalhador São-Carlense, 400, São Carlos 13566-590, SP, Brazil; 2 Departamento de Química, Universidade Federal de São Carlos, Rodovia Washington Luis, km 235, São Carlos, 13565-905, SP, Brazil

**Keywords:** chitosan, naproxen, chitosan salts, epichlorohydrin, dissociation equilibrium

## Abstract

Salts of naproxen (NAP) with chitosan (CTS) and reticulated chitosan (CEP) were prepared under optimized conditions to maximize the yield of reaction. The objective was to evaluate the dissociation in water, which can guide studies of release of the drug from biopolymeric salts in pharmaceutical applications. Higher salification was found after 24 h of reaction at 60 °C in a molar ratio 1:1.05 (CTS:NAP, mol/mol), resulting in a degree of substitution (DS) of 17% according to ^13^C NMR, after neutralization of the –NH_2_ group of the biopolymer by the carboxylic group of the drug. The presence of NAP salt is evidenced by FTIR bands related to the –NH_3_^+^ group at 856 cm^−1^, a decrease in crystallinity index in XRD diffractograms as well as changes in mass loss ratios (TG/DTG/DTA) and increased thermal stability of the salt regarding CTS itself. The CEPN crosslinked salt presented a DS = 3.6%, probably due to the shielding of the –NH_2_ groups. Dissociation studies revealed that at pH 2.00, dissociation occurred faster when compared to at pH 7.00 in the non-reticulated salt, while the opposite was observed for the reticulated one.

## 1. Introduction

Chitin (CT) is a structural polysaccharide extracted from exoskeletons of crustaceans (crabs, shrimps, lobsters), squid gladii and in smaller amounts from insects and fungal cell wall [1,2,3,4,5]. Chitosan (CTS) is obtained by the deacetylation reaction of chitin in alkaline media, resulting in a natural copolymer composed of 2-amino-2-deoxy-D-glycopyranose and 2-acetamido-2-deoxy-D-glycopyranose units randomly linked by glycosidic bonds *β*(1 → 4) [6,7,8,9].

Physicochemical and biological properties of CTS have garnered attention due to its polycationic character, biocompatibility, biodegradability, mucoadhesiveness and antibacterial activity, among other interesting properties [10,11]. These characteristics are related to its degree of acetylation (DA) and molar mass. CTS had been characterized by techniques such as ^1^H and ^13^C nuclear magnetic resonance (NMR), Fourier transform infrared spectroscopy (FTIR), ultraviolet–visible spectroscopy (UV–vis), thermogravimetry coupled to Fourier transform infrared spectroscopy (TG–FTIR), differential scanning calorimetry (DSC) and elemental analysis [1,2,12,13,14], and its average molar mass can also be assessed by viscosimetry and gel permeation chromatography (GPC) [15,16].

Amine and hydroxyl groups present in CTS chains enable several modifications including N-alkylation, N-acetylation, crosslinking, preparation of salts and Schiff bases and N-carboxylation, among many others [12,17,18]. Due to the possibility of reactions with different groups, CTS modifications have been studied, and the resulting products used in chemical, biomedical, pharmaceutical, biological and other areas of human knowledge [10,11,19,20,21].

Ionic interaction became an attractive issue in the study of dissociation of matrixes formed by CTS and other molecules that are sensitive to changes in the pH of the medium. In this sense, the formation of CTS salts has been an alternative in the preparation of systems that respond to changes in chemical environments [22,23]. Such systems are widely developed for use in biomedical and pharmaceutical areas, including drug delivery [24,25].

Thus, salts of ibuprofen [26] and indomethacin [27] anti-inflammatories with CTS were prepared with CTS of different molar masses, and the effect of the polymeric chain size in the interaction and solubility were evaluated. Salts of CTS micro/nanoparticles with insulin, sodium diclofenac and acetyl salicylic acid were prepared and evaluated regarding controlled release from the biopolymeric matrix [22], and it was also done for other inorganic and organic acids [28].

Naproxen (NAP) ((+)-(*S*)-2-(6-methoxynaphthalen-2-yl) propionic acid) is a non-steroidal anti-inflammatory drug (NSAIDs) widely prescribed in the whole world. It presents anti-inflammatory, anti-thermic and analgesic action related to the inhibition of cyclo-oxygenase enzymes COX1 and COX2, which catalyze the production of prostaglandins, physiologically active lipids that induce inflammation [29,30].

NAP dissolution was evaluated in CTS–NAP systems prepared by physical mixtures aiming for the better dissolution of NAP in Caco-2 type cells, concluding that a higher polymer-to-drug ratio led to an increasing solubility at aqueous medium [31,32]. When prepared from different drying methods in which κ-carrageenan and hydroxypropylmethylcellulose were added aiming to follow the release of drug, it was found that the matrix with κ-carrageenan presented higher swelling capacity, consequently decreasing NAP release [33]. In a CTS–PVP polymer matrix, the addition of PVP improved the process of NAP release [34].

The effect of glutamate and hydrochloride salts of CTS in the dissolution rate of NAP in Caco-2 type cells was evaluated, resulting in the conclusion that pulverization was more effective than other procedures, improving the permeability of NAP in the cell layer, and the effect of electrolytes in NAP dissolution was also investigated [35], which showed on the other hand that electrolyte-containing calcium cations slowed down the release of NAP [36].

CTS hydrogels loaded with NAP have also been prepared and evaluated regarding their effect on releasing the drug in dentin dental tissues, presenting a synergistic effect that improved NAP action when CTS is associated with dentin [37] and nanocapsules of CTS–NAP ionic gelation that presented antibacterial action [38].

However, despite the importance of NAP and its association with CTS, there is a lack in literature regarding a better understanding of ionic interaction between free amine groups in CTS chains and carboxylic groups from NAP. Such studies can be useful in the comprehension of the dissociation equilibrium of NAP from the polymeric matrix when exposed to different pH media in biological environments such as stomach and intestines. The dissociation equilibrium of the drug–polymer matrix is fundamental for future applications in which this system is used as a carrier in delivery of the drug as well as in its dissolution.

In this work, the synthesis of CTS salts with NAP is proposed under different parameters such as reaction time, temperature and molar ratio of reactants. Final products were characterized by spectroscopy techniques and thermoanalytical methods. Moreover, an investigation of the dissociation of NAP from chitosan salt at pH 2.00 and pH 7.00 simulating the stomach and intestinal chemical environments was performed in order to propose a mechanism for dissociation. The objective was to evaluate the dissociation in water, which can guide studies of release of the drug from the biopolymeric salt in pharmaceutical applications.

## 2. Results and Discussion

NAP–CTS salts were prepared under different conditions as detailed described in the experimental section. Table 1 presents a description of the samples and the abbreviations used all along this discussion and summarizes the experimental parameters used to obtain each salt used in the present study.

### 2.1. ^13^C NMR Spectroscopy

**Figure 1:**
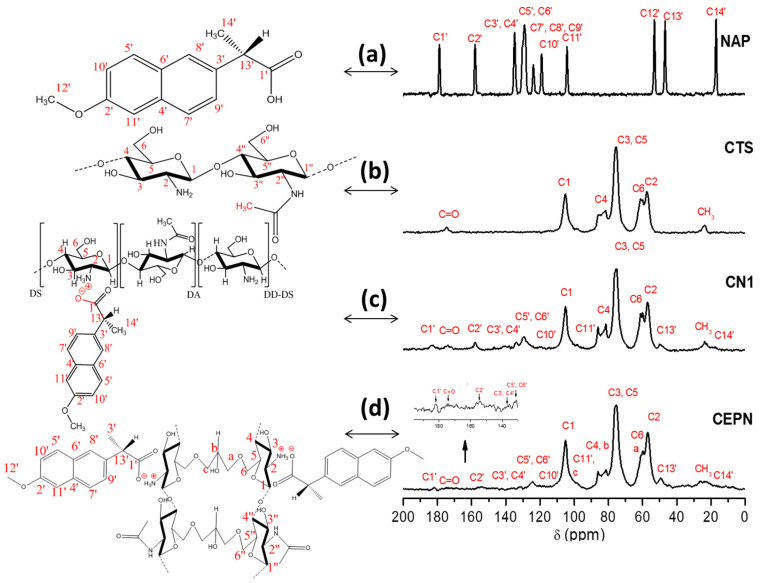
^13^C NMR spectra of reagents and reaction products: (**a**) NAP, (**b**) CTS, (**c**) CN1, (**d**) CEPN, and the structural formula identifying the carbon atoms for assignments.

^13^C solid state NMR spectra were used to evaluate the formation of salts as well as to estimate the relative amount of NAP present in the CTS matrix calculated from the degree of substitution, $\bar{DS}$. Figure 1 depicts ^13^C NMR spectra of NAP and CTS starting reagents, CN1 and the salt formed from NAP and CTS crosslinked with epichlorohydrin (CEPN), with the respective assignments. Table 2 presents the attributions for the main peaks observed in the CTS [39] and NAP [40] spectra, according to the literature, and also the peaks observed in the spectra of salts, based on the spectra of the precursors.

When the spectra of the products and CTS were compared, changes in the values of chemical shifts of the starting material could be observed. The peak related to the C1′ of NAP shifted from 178.9 to 183.7 ppm. The peak of C13′ shifted from 46.8 to 49.4 ppm, and that of C14′ slightly shifted from 17.0 to 17.6 ppm, after the reaction. These changes suggest that the formation of the salt was successful. Additionally, a strong interaction between NAP and CTS could be observed due to the intense broadening of NAP signals.

The degree of substitution $\bar{DS}$ was calculated using Equation (1), which relates the integrated areas of C1′ of NAP in relation to the C1 of the glycopyranoside ring from CTS, considered as a unit. $\bar{DS}$ also corresponds to the yield to each synthesis. This calculation has been proposed, in a similar idea, for $\bar{DS}$ determination in biopolymeric Schiff bases [2].

(1) $$\bar{DS}=\frac{{C1}^{\prime }}{C1}\times 100$$

From this equation, $\bar{DS}=$ 17% was found as a result of the best reaction condition. Table 3 summarizes the $\bar{DS}$ of each salt formed under different sets of reaction conditions compared to CTS starting reagents and CEPN products. It seems that the mol ratio used plays an important role in the reaction yield associated with the temperature.

According to the data in Table 3, the highest degree of substitution was observed for the salt CN1, whose ^13^C NMR spectrum is presented in Figure 1c. CN1 was obtained under the reaction condition that resulted in the highest salification yield.

The crosslinked CTS was prepared to evaluate the effect of the crosslinked polymer chains in the interaction of the biopolymeric matrix with the drug and its dissociation in the salt called CEPN, whose spectrum is also presented in Figure 1 together with those of the starting reagents and CN1 salt. The formation of CEPN salt was suggested due to the presence of peaks at 182.4 ppm, 153.8 ppm and 115–150 ppm. The increasing in the intensity of the peak at 59.8 ppm evidenced the CTS reticulation by epichlorohydrin [39,41].

The resulting degrees of substitution for CN1 (17%) and CEPN (3.6%) salts revealed a higher interaction between CTS and NAP in the salt CN1 when compared to CEPN, leading to the conclusion that the reticulation process seems to result in a lower exposure of amine groups present in CTS, decreasing its potential of reaction with carboxyl groups of NAP.

### 2.2. Infrared Spectroscopy

**Figure 2:**
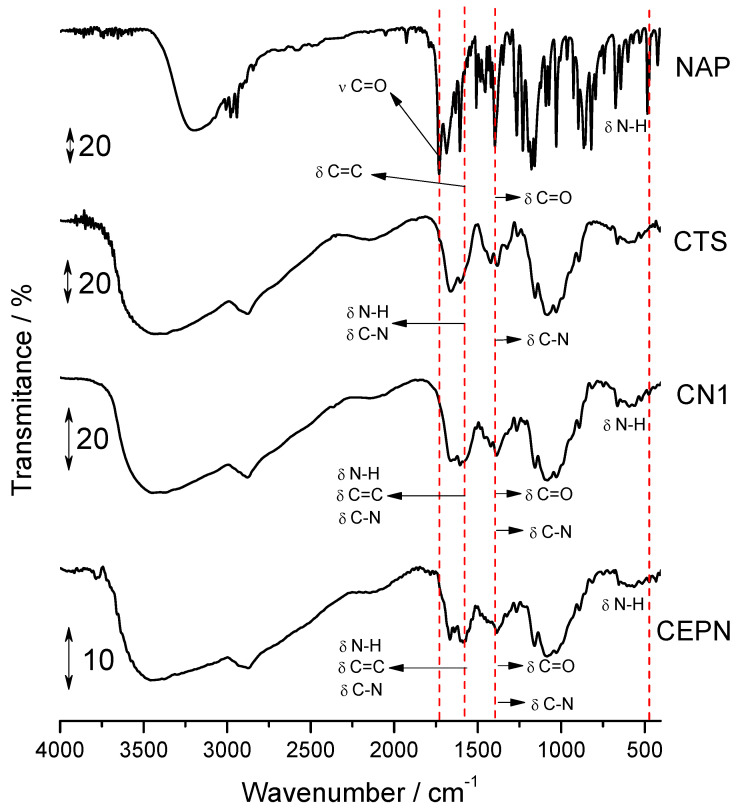
FTIR spectra of CTS, NAP, CN1 and CEPN.

Figure 2 presents FTIR spectra of the starting reagents and final products, with the highest $\bar{DS}$.

The main bands observed in these spectra are summarized in Table 4, and they are attributed according to the literature for CTS [12,15,42,43] and NAP [44,45]. The CN1 FTIR spectrum showed a profile similar to that of CTS. However, in the region of 1800–400 cm^−1^, differences could be observed when compared to the spectra of the starting reagents. At 1606 cm^−1^, the bands referent to axial deformation of the N–H group were intensified, once the band of the C=C group overlapped; there were intensifications of other bands at 1552 and 1382 cm^−1^; and the absence of a 1728 cm^−1^ signal related to the carboxylate ion band, corroborating the salt formation. At 817 cm^−1^ an intensification of the C–H band was observed, in addition to 484 cm^−1^, in which a slight band was attributed to the groups -NH_3_^+^, and is characteristic of the protonate amine group [43].

In this case, the use of a proper mol ratio associated with a higher temperature resulted in the largest reaction extension, and the FTIR spectra were fundamental to present the salt formation as mainly represented by the reduction of the carbonyl signals from NAP at *c.a.* 1730 cm^−1^ bands and the appearance of the –NH_3_^+^ signals at ca. 480 cm^−1^, which was observed in all cases, but with highest intensity in CN1 and CEPN samples.

For the CEPN FTIR spectrum, a decrease in the intensity of the band was observed at 3400 cm^−1^ referent to O–H bond stretching, resulting from the reaction of the O–H group of the CTS chain with crosslinked agents [46]. Moreover, there was an increase in the intensity of the band already existing at 2900 cm^−1^ when compared to CN1. The bands in the 1700–1250 cm^−1^ region demonstrated different profiles regarding the spectra of CN1 and CTS, justifying the presence of NAP and epichlorohydrin and thus confirming the formation of the crosslinked salt.

### 2.3. UV–Vis Diffuse Reflectance Spectroscopy

To obtain the absorption spectra of UV–Vis in diffuse reflectance mode, the transformation of Kubelka–Munk was applied, once the data were generated as reflectance signals [47].

According to the absorption spectra present in Figure S1a (Supplementary Material), CTS absorbed at 210 and 266 nm related to N-acetyl-glucosamine and glucosamine groups, respectively [48,49]. These transitions may have been n–π* transitions of C=O, NH_2_ and OH groups. NAP molecules absorbed at 241, 273 and 332 nm. This bands can be assigned to π–π* transitions from the conjugated π system of the ring, in addition to the n–π* transition of C=O, NH and OH groups [50]. In the CN1 spectrum, the band at 210 nm was less intense, suggesting that the electron pair of NH_2_ group interacts with the proton from the carboxylic acid of the drug. Furthermore, an increase in the intensity of the band at 333 nm was observed due to the overlap of absorption bands of CTS and NAP.

For CEPN (Figure S1b, Supplementary Material), an increase in the intensity of absorption bands at 270 and 330 nm could be observed, evidencing the presence of epichlorohydrin and NAP in the CTS matrix.

### 2.4. X-ray Diffraction

Figure S2 (Supplementary material) presents X-ray diffractograms of CTS, NAP, CN1 and CEPN. CTS is a semicrystalline carbohydrate that presents two characteristic regions in its diffractogram. The peak observed at 2θ = 10° is related to amorphous region of biopolymer, while the peak at 2θ = 20° is characteristic of the crystalline portion [51]. It is associated with inter and intramolecular hydrogen bonds between NH_2_ and OH groups present in the biopolymeric chain. From the intensity of these two peaks, the crystallinity index of CTS and their salts could be calculated according to Equation (2) [52]:

(2) $$Id\;\left(\%\right)=\frac{\left(I_{110}-I_{am}\right)}{I_{am}}\times 100$$

in which *I*_110_ is the relative intensity of the peak signal at ca. 2θ = 20°, and *I_am_* is the intensity of the amorphous portion at ca. 2θ = 10°.

The starting CTS used in this work presented 61% crystallinity. After the reaction with NAP, the value decreased to 49% and 58% for CN1 and CEPN, respectively. This means that the ionic interaction between amine and carboxyl groups present in the structure of CTS and NAP reduced the number of hydrogen bonds, pointing to formation of the salts. The higher crystallinity in CEPN agrees with the lower $\bar{DS}$ in this case. In addition, a small shoulder appeared at 22°, suggesting modification on the crystalline portion due to presence of NAP in the biopolymer matrix.

The lower crystalline index observed in the salt when compared to the biopolymeric matrix can suggest that the hydrogen bonds that are responsible for the semi crystallinity of CTS itself decreased once the –NH_2_ free groups were compromised by the salt formation.

### 2.5. Thermal Analysis

**Figure 3:**
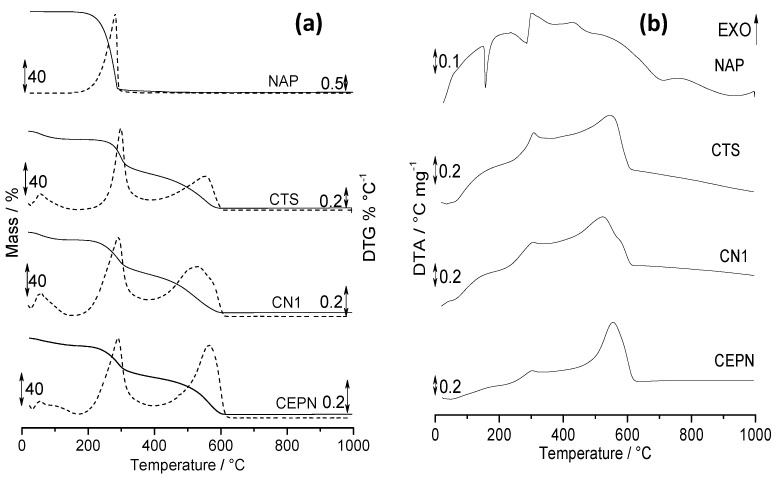
(**a**) TG/DTG and (**b**) DTA curves of NAP, CTS, CN1 and CEPN obtained in dynamic air atmosphere, flowing at 50 mL min^−1^, sample mass 7.0 ± 0.1 mg, in open *α*-alumina sample holders, with a heating rate of 10 °C min^−1^.

Figure 3 presents TG/DTG (Figure 3a) and DTA (Figure 3b) curves of CTS, NAP, CN1 and CEPN obtained under the conditions described in the experimental section. Quantitative data related to the events, temperature ranges, mass loss and final residue can be verified in Table 5. In this Table the ratios between the second and first mass losses are also presented, as well as peak temperatures from DTA curves.

In agreement with previous reports [30], NAP remained thermally stable up to 152.5 °C and then presented two steps of mass losses. The first one corresponded to the decomposition of the drug, followed by the second one, which related to the burn of residual carbonaceous material, as observed in the TG/DTG curve. Additionally, in agreement with the literature [30], the DTA curve presented a sharp endothermic peak referent to melting at 156.7 °C, followed by one endo and another exothermic peak due to dehydration and degradation of the drug and burning of carbonaceous material, respectively.

The events observed in themogravimetric curves of CTS under air are well described in the literature [2,12]. The biopolymer usually presents one step of mass loss assigned to dehydration, in addition to second and third steps that are referent to the degradation of acetylated and deacetylated chains and burning of carbonaceous material, as observed in its TG/DTG curves (Figure 3a). The DTA curve showed an endothermic peak characteristic of dehydration and two others related to biopolymer decomposition [2,12].

TG/DTG curve of both CN1 and CEPN presented profiles similar to those observed for the CTS curve. However, it can be seen that the salt formation promoted shifts in temperature ranges as well as changes in mass losses according to Table 5. It suggests that these salts present different thermal properties from starting reagents, confirming the modification. Three events were observed, which were also attributed in the same way to the CTS curve. Furthermore, peaks present in the DTA curve were attributed to dehydration and decomposition with small differences. For CN1, the exothermic peak at 522.0 °C presented a shoulder, evidencing that the burning of this material appeared to split in two. For CEPN, the peak at 553.5 °C presented the same profile of CTS; however it seemed to be more defined.

The ratio between the second and first mass loss steps in the TG curves of CTS was equal to 1.0. However, when taking into account the same ratio for CN1 and CEPN salts, this value was higher than 1.0, suggesting that the decomposition of the salt portion of the polymer and NAP present seemed to decomposed in the second step.

Figure S3 (Supplementary Material) presents DSC curves of CTS and NAP as well as the products of reaction of CN1 and CEPN. In the DSC curve of NAP could be observed the presence of a sharp endothermic peak at 156.7 °C relative to the drug melting [30], while the CTS curve presented one broad endothermic peak at 89.0 °C assigned to the loss of water from polymeric structure of CTS [14]. Thus, when comparing the curves of the reaction products CN1 and CEPN with NAP and CTS, a similar broad endothermic peak as in CTS was verified, with slight changes in the temperature range of the event and peak profiles. However, the melting peak relative to NAP was not observed, suggesting an interaction between the pharmaceutical and the biopolymer.

Thus, thermal results evidenced a change in the thermal behavior of NAP after reaction with CTS to form CN1 and CEPN. The same evidence appeared in DTA curves (Figure 3b) and corroborated ^13^C NMR and FTIR spectra regarding the formation of CN1 and CEPN salts.

### 2.6. Dissociation Study Using High Performance Liquid Chromatography (HPLC)

**Figure 4:**
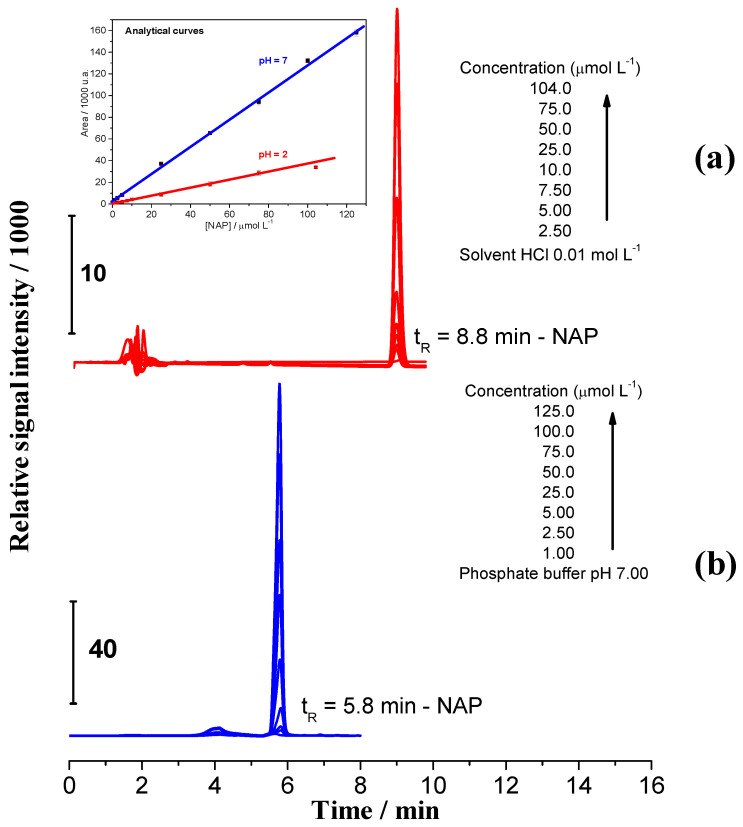
Chromatograms of NAP in different concentrations (**a**) at pH 2.00, details inserted of the signal referent to NAP in HCl 0.01 mol L^−1^ solution; (**b**) at pH 7.00, details inserted of the signal referent to the phosphate buffer pH 7.00.

The dissociation of naproxen from CN1 and CEPN salts was studied at pH 2.00 (simulating the stomach) and 7.00 (simulating the intestine) environments by HPLC. Initially, an analytical curve was established to evaluate the concentrations of samples, and the analytical signals were measured with a UV–vis detector at 270 nm. Figure 4 presents the chromatograms in both HCl 0.01 mol L^−1^ and phosphate buffer pH 7.00, used as blanks, to obtain the analytical curve.

In Figure 4a it is possible to see lower intensity signals at t_R_ 2 min, attributed to HCl in solution, followed by those related to NAP at t_R_ 8.8 min. In Figure 4b, the low intensity signals are referent to phosphate buffer at t_R_ 4.0 min and those at t_R_ 5.8 min are referent to NAP. The difference in retention times was attributed to the protonation equilibrium once the *pKa* of the pharmaceutical was 4.2 [29].

At pH 2.00, NAP was preferably in the protonated form, making it less soluble when compared with NAP at pH 7.00 solution, in which it was preferably in the basic form, increasing the solubility and lowering its retention inside the column.

Using the peak areas for the different concentrations in NAP at pH 2.00 and 7.00, it was possible to obtain the analytical curves, as seen in the insets of Figure 4. The analytical parameters related to the linear dynamic ranges are summarized in Table S1 (Supplementary Material).

The dissociation studies were performed using 0.10 g L^−1^ solutions of CN1 and CEPN salts suspended in both HCl solution pH 2.00 and phosphate buffer pH 7.00 under stirring, at 37 °C. Figure S4 (Supplementary Material) presents the chromatograms of aliquots of these solutions taken after 11, 77 and 155 min.

For the chromatogram of CN1 in HCl pH = 2 (Figure S4a—Supplementary Material), it was possible to observe two signals at retention times of 0.6 min and at 8.7 min. These two signal increased over time. The signals at 0.6 min were attributed to the solubilized fraction of CTS once it had a *pKa* of approximately 6.5, while the signal at higher retention time was referent to NAP dissociated from the salt.

For the chromatogram of CEPN in HCl pH = 2 (Figure S4b—Supplementary Material), the signal referent to CTS was not observed, suggesting a lower solubility of crosslinked material even in such an acidic medium.

Chromatograms of CN1 and CEPN salts suspended in phosphate buffer pH 7.00 are presented in Figure S5 (Supplementary Material). In both cases, the NAP peak appeared at t_R_ 5.80 min, in agreement with chromatograms in Figure 4. The neutral medium avoided CTS solubilization, while the signal observed at 4.00 min was attributed the phosphate buffer.

**Figure 5:**
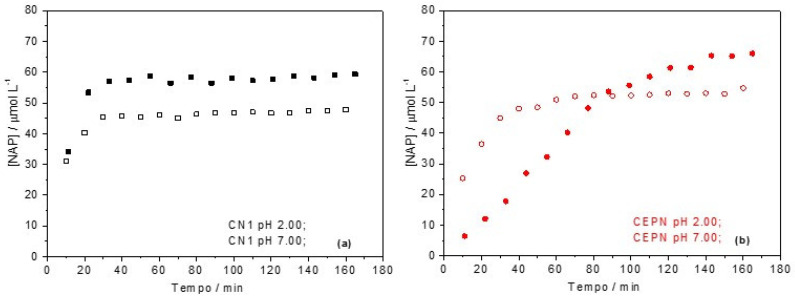
Dissociation profiles of NAP as a function of time in different media: (**a**) CN1 pH 2.00 (filled black) and 7.00 (leaked black); (**b**) CEPN pH 2.00 (filled red) and pH 7.00 (leaked red).

Using the data from the analytical curve (Table S1, Supplementary Material) and the peak areas in chromatograms for NAP solution aliquots taken after different times, it was possible to obtain the dissociation profiles, which are presented in Figure 5.

In Figure 5a, similar profiles can be observed for CN1 at pH 2.00 and 7.00, with equilibrium being reached after 35 min. However, the final mass of NAP in solution was 22% lower at pH 7.00 than at pH 2.00, probably due to protonation of the pharmaceutical that had a difficult interaction with the biopolymer matrix.

Figure 5b described the dissociation profiles of the CEPN salt at pH 2.00 and 7.00, which are clearly different. While at pH 7.00 a similar profile as that verified for CN1 could be observed, at pH 2.00 the CEPN presented a slower dissociation, resulting in a larger equilibrium concentration, but only after 140 min when a plateau was reached. Data obtained from the dissociation profiles are summarized in Table 6.

**Figure 6:**
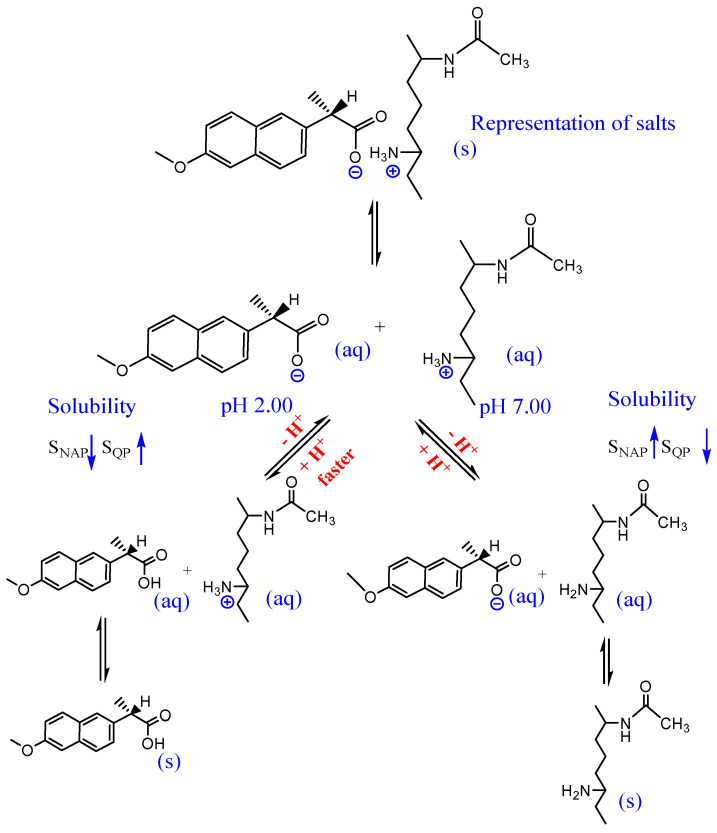
Proposal of dissociation mechanism of NAP from salts.

Therefore, from the dissociation of NAP at pH 2.00 and 7.00 by HPLC, a mechanism of dissociation can be proposed, such as that presented in Figure 6.

According to the Le Chatelier principle, when CN1 and CEPN salts were submitted to the dissociation at pH 2.00, an equilibrium shift occurred towards the protonation of the carboxylic group present in NAP (*pKa* = 4.2), as well as the protonation of the amine group of CTS (*pKa* = 6.3), leading to dissociation of NAP and eventual precipitation, while CTS remained in solution. In neutral media of pH 7.00, the equilibrium was shifted, CTS was not protonated and presented a lower solubility, while the NAP remained deprotonated, with a higher trend to be released in solution.

However, it was observed that NAP was readily dissociated from CTS with both pH values, staying in solution at higher concentrations at pH 2.00. Furthermore, at pH 7.00, the CTS was not fully protonated (*pKa* = 6.3), with part of the drug still linked to the biopolymeric chains.

In the case of the crosslinked salts at pH 7.00, dissociation occurred in an extension similar the CN1. However, at pH 2.00, for CEPN there was a higher dissociation when compared to pH 7.00 and also the different pH values tested for CN1, but the time to reach equilibrium was higher than for the other salts. The slower dissociation held the NAP in the crosslinked biopolymer matrix for a longer time.

## 3. Material and Methods

### 3.1. Reagents and Solutions

All reagents were of analytical grade (PA) and used as received, except for chitosan, which was purified as described in the next section. All solutions were prepared using ultrapure H_2_O obtained from a D13321 Barnsted^TM^ EASYpure Rodi^®^ system (Thermo Scientific, Dubuque, IA, USA), conductivity 18.2 Ω cm^−1^, after pre-treatment in an OS 10LZ reverse osmosis system (Gehaka, São Paulo, Brazil).

Reagents used included glacial acetic acid and disodium hydrogen phosphate (J.T. Baker, Xalostoc, Edo. de México, Mexico); hydrochloric acid (Panreac, Castellar del Vallès, Spain); ammonium hydroxide (Exodo, Sumaré, Brazil); sodium hydroxide and potassium dihydrogen phosphate (Spectrum, New Brunswick, NJ, USA); absolute ethyl alcohol (Synth, Diadema, Brazil); acetonitrile, phosphoric acid (Mallinokrodt, Staines-upon-Thames, UK); naproxen 99.5% (Pharmanostra, Campinas, Brazil); deuterium oxide, epichlorohydrin ≥ 99.0% and chitosan low molar mass ($\bar{M}v$ = (4.3 ± 0.4) 10^4^ Da and degree of deacetylation ($\bar{DD}$) of 89.9%) (Sigma Aldrich, St Louis, MO, USA).

### 3.2. Chitosan Purification

The purification of CTS was performed as proposed by Signini and coworkers [53,54], in which 15 g of commercial CTS was added to a solution of acetic acid 3% (*v*/*v*) and maintained under stirring for 18 h at room temperature. After that it was filtered three times in a sintered disk funnel. Then, concentrated NH_4_OH was added to the resulting mixture and stirred for 1 h. Precipitated CTS was neutralized, filtered and washed with water until neutral pH, and it was finally washed with ethanol. CTS was dried at 40 °C under low pressure and kept in a desiccator [53,54]. After that, the mol mass of CTS was determined by viscosimetry as ($\bar{M}v$ = (1.6 ± 0.4) 10^4^ Da).

### 3.3. Reaction of Chitosan and Naproxen

**Figure 7:**
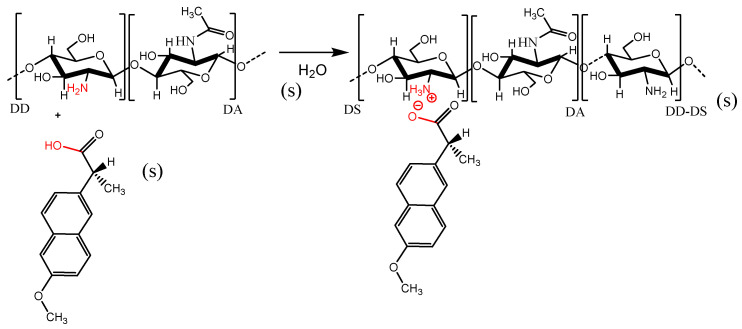
Scheme proposed for the reaction between CTS and NAP for preparation of the CN salt. Optimized reaction parameters: temperature 40/60 °C; reaction time 24/48 h; CTS:NAP mol ratios of 1:1.05 and 1:1.50 (*mol*/*mol*).

Different amounts of NAP (see Table 1) were suspended in 300 mL of ultrapure water, and purified CTS was added to this solution. The system was maintained under magnetic stirring (520 rpm) and controlled heating. The final product was filtered, washed with ethanol to remove the excess of reactants and dried at 40 °C under vacuum. The effects of reaction parameters of synthesis such as reaction time (24 and 48 h), temperature (40 and 60 °C) and mol ratio (1:1.05 and 1:1.5 CTS:NAP *mol/mol*) were evaluated in order to obtain the best yield. Table 1 presents the reaction conditions used, while Figure 7 represents a reaction scheme.

### 3.4. CTS Reticulation

In this case, the procedure used was adapted from Silva and coworkers, in which 700 mg of CTS was suspended in 300 mL of ultrapure water under stirring, and pH was adjusted to 10 with NaOH 0.01 mol L^−1^. After that, 166 μL of epichlorohydrin crosslinking agent was added to the solution. The reaction mixture was kept for 2 h at 40 °C under stirring. The final product was filtered, washed with ethanol and dried at 60 °C under vacuum for 2 h [46]. The salt prepared from reticulated CTS was synthetized under the optimized conditions established in the previous section, and it was called CEPN.

### 3.5. Study Regarding NAP–CTS and NAP–CEPN Salt Dissociation by High Performance Liquid Chromatography (HPLC)

The determination of NAP concentration dissociated from the CTS matrix by HPLC in both pH 2 and 7 was based on previously described procedures [55,56]. For dissociation in pH 2.00, H_3_PO_4_ 0.010 mol L^−1^:acetonitrile (55:45 *v*/*v*) mobile phase flowing at 1.5 mL min^−1^ was used and resulted in a retention time of 8.7 min for NAP. In pH 7.00, the mobile phase was a phosphate buffer pH 7.00:acetonitrile (55:45 *v*/*v*) mixture, resulting in a retention time of 5.8 min for NAP.

The chromatographic procedures were performed in an LC-20AD Shimadzu system, equipped with a C18 Phenomenex column (5.0 µm, 250 mm, 4.6 mm) and injections of 20.0 µL. The column and the samples were kept at 37.0 °C, simulating the body temperature.

Analytical curves in the concentration range 1.00 × 10^−6^–1.25 × 10^−4^ mol L^−1^ in both cases were prepared by diluting appropriate amounts of NAP stock solutions in HCl 0.010 mol L^−1^ or phosphate buffer for studies in pH 2.00 and 7.00, respectively. For this, a stock solution in pH 2.00 was prepared by dissolving 1.20 mg of NAP in 12.0 mL of ethanol and completed to the final volume of 50.0 mL with HCl 0.010 mol L^−1^. In pH 7.00, a stock solution was prepared by dissolving 5.00 mg of NAP in 50.0 mL of phosphate buffer pH 7.00.

Finally, for dissociation studies, 5.00 mg of NAP–CTS and NAP–CEPN salts were suspended in 50.0 mL of HCl 0.010 mol L^−1^ or phosphate buffer for pH 2.00 and 7.00, respectively, and kept under magnetic stirring at 37.0 °C during the entire experiment. Aliquots of 20.0 µL were withdrawn at each 10 min and injected in the chromatographic system. Before each injection, the aliquots were filtered through a 0.45 µm porous size membrane (Chromafil, PETY-45/15 MS). The monitoring was performed until 180 min, the time at which changes in NAP concentrations were no longer observed.

### 3.6. Characterization

#### ^1^H and ^13^C NMR Spectroscopy

^1^H NMR spectra were obtained in a model 400/54 premium shielded Agilent Technologies 9.4 Tesla (400 MHz to frequency hydrogen) spectrometer with an ^1^H detector. Thus, 10 mg samples were solubilized in 1.0 mL of D_2_O and 10.0 μL of HCl and kept under stirring for 18 h at 25 °C. Such a spectrum was used to determine the DD of chitosan and was obtained only for this biopolymer.

^13^C solid state NMR spectra were obtained in a Bruker Advance III-400—9.4 T (399.94 MHz for ^1^H) spectrometer, equipped with a 4 mm MAS probe-head. Powdered samples were packed into a 4 mm zirconia rotor and spun at 5 kHz of spinning speed. The spectra were acquired under cross polarization conditions using a CPTOSS (cross polarization with total sideband suppression) pulse sequence. For these experiments, 256 and 2048 scans for NAP and NAP–CTS samples were collected, respectively, using 3 s of recycle delay and 3 ms of contact time.

### 3.7. Infrared Spectroscopy (FTIR)

Vibrational spectra in the infrared region were recorded in an IRAffinity-1 FTIR spectrophotometer (Shimadzu, Kyoto, Japan), operated in the 4000–400 cm^−1^ range, with 4 cm^−1^ resolution and 32 acquisition scans. Samples were prepared from mixtures of 5.0 mg of sample and 95.0 mg of KBr, pressed as pellets.

#### UV–Vis Diffuse Reflectance Spectroscopy

Spectra were obtained in an UV–visible–NIR model UV 3600 spectrometer (Shimadzu, Japan), equipped with a module for solid state analysis, in diffuse reflectance mode, in the wavelength range of 200–800 nm. The conversion of reflectance to absorbance was performed upon application of Kubelka–Munk transformation [46].

### 3.8. X-ray Diffraction (XRD)

The diffractograms were obtained in a D8 Advance Bruker X-ray diffractometer, equipped with a probe radiation of copper (λ = 1.54 Angstrom) and position sense detector. The diffractograms were acquired in the θ/2θ mode coupled with a pass of 0.02° and accumulation time of 0.5 min.

#### Thermal Analysis

TG/DTG/DTA curves were obtained using a simultaneous TG/DTA SDT-Q600 module managed by Thermal Advantage software^®^ for Q Series v.5.5.24 (both from TA Instruments, New Castle, DE, USA). The measurements were performed under dynamic dry air atmosphere, flowing at 50 mL min^−1^, using a sample mass of 7.0 ± 0.1 mg, a temperature range of 25–1000 °C and a heating rate of 10 °C min^−1^ in open *α*-alumina sample holder.

DSC curves were recorded in a Q10 differential scanning calorimetric module controlled by Thermal Advantage Series^®^ software v.5.5.24 (both from TA Instruments) using a sample mass of 5.0 mg ± 0.1 mg, at a heating rate of 10 °C min^−1^, under dynamic N_2_ atmosphere, flowing at 50 mL min^−1^, from 25 to 210 °C, in closed alumina sample holder with a pin hole (ø = 0.7 mm) in the center of the lid.

## 4. Conclusions

CN and CEPN salts were successfully prepared from CTS and NAP, and an enhanced yield was found under optimized reaction conditions including time, temperature and molar ratio. According to ^13^C NMR, the crosslink process influences the disposition of amine groups in chains of CTS, decreasing its availability for reaction. In the FTIR spectra, the signal related to protonated ammonium was observed, while the lower crystallinity in the reaction products, observed in XRD diffractograms, strongly suggested that the free –NH_2_ groups were compromised with the salt formation, reducing the hydrogen bonds that allow the semi crystalline character of CTS itself. The products were more thermaly stable than expected for a salt regarding the starting biopolymer. Despite the decrease in dissociation speed in crosslinked material, higher amounts of NAP could be seen in solution after all. The slower dissociation, associated with a higher free drug concentration at the end of the dissolution process in CEPN, may indicate an increase in drug retention in this system by reducing the loss of drug during ingestion process and favoring its liberation in the intestines.

## Figures and Tables

**Table 1:** Parameters used in the preparation of the salts.

Product Code	Ratio CTS:NAP (mol:mol)	Reaction Time (h)	Temperature (°C)
CN1	1.00:1.05	24	60
CN2	1.00:1.05	24	40
CN3	1.00:1.05	48	60
CN4	1.00:1.50	24	60
CN5	1.00:1.50	48	60
CN6	1.00:1.50	48	40

**Table 2:** Assignment of the chemical shift of the ^13^C NMR spectra of NAP, CTS, CN and CEPN salts.

Chemical Shifts (δ) (ppm)																	
	C1′	C=O	C2′	Naphthalene Ring	C1	C11′	(c)	(b)	C4	C3,C5	C6	(a)	C2	C12′	C13′	CH_3_	C14′
NAP	178.9	-	158.1	115.0–150.0	-	104.2	-	-	-	-	-	-	-	53.1	46.8	-	17.0
CTS	-	174.7	-	-	105.2	-	-	-	81.6	75.5	61.1	-	57.5	-	-	23.7	-
CN1	182.7	173.9	157.9	115.0–150.0	105.2	98.0	-	-	81.4	74.5	59.9	-	57.1	-	50.0	23.7	18.3
CN2	183.1	174.7	158.1	115.0–150.0	105.2	97.6	-	-	81.5	75.6	59.9	-	57.1	-	49.4	23.7	20.4
CN3	182.9	174.6	157.8	115.0–150.0	105.2	99.5	-	-	81.6	75.5	60.0	-	57.2	-	49.6	23.6	19.2
CN4	182.6	174.6	157.9	115.0–150.0	105.2	98.0	-	-	81.5	75.5	60.0	-	57.2	-	49.9	23.8	21.1
CN5	182.6	175.0	158.1	115.0–150.0	105.2	97.7	-	-	81.5	75.5	60.0	-	57.2	-	49.5	23.6	20.6
CN6	182.8	174.5	158.1	115.0–150.0	105.2	97.6	-	-	81.6	75.6	60.0	-	57.2	-	49.4	23.9	17.6
CEPN	182.4	173.8	153.8	115.0–150.0	105.1	98.1	100.2	86.1	81.4	75.42	60.9	59.8	56.9	-	49.3	26.3	22.6

**Table 3:** Relative areas of ^13^C NMR peaks of carbons C1 and C1′ of the salts.

	Sample *	CTS	CN1	CN2	CN3	CN4	CN5	CN6	CEPN
Parmeter *									
C1		1.00	1.00	1.00	1.00	1.00	1.00	1.00	1.00
C1′		-	0.17	0.15	0.12	0.12	0.12	0.11	0.036
$\bar{DS}$ **(%)**		-	17	15	12	12	12	11	3.6

* Abbreviation refers to Table 1 for reactants and products; C1 and C1′, carbon atoms as defined in Figure 1; $\bar{DS}$ (%), the degree of substitution from Equation (1).

**Table 4:** Assignment of the bands observed in the FTIR spectra of NAP, CTS, CN1 and CEPN.

Samples	ν_O-H_/ν_N-H_	ν_C-H_ asym./sym.	ν_C=O_ Carboxyl	δ_C=O_ Amide I	δ_N-H_ Amine	δ_C=C_ Naphth. Ring	δ_C-N_ Amide	δ_C-N_ Amide II	*δ_CH_3__/δ_COO^−^_*	CH_3_ Amide	δ_C-N_ Amide III	ν_C-O_ Ether	δ_C-H_	ν_C-O-C_ β(1→4)	δ_C-OH_	C-H Glucopy. Ring.	δ_C-H_	δ_N-H_ Ammonium
NAP	3196	2976 2939	1728	-	-	1602	-	-	1394	-	-	1265	1176	-	-	-	819	484
CTS	3600–3100	2917 2877	-	1662	1604	-	1552	1435	-	1382	1325			1159	1089 1029	891		
CN1	3600–3100	2937 2870	-	1664	1606	1606	1573	1425	1381	1381	1321	1269	1209	1157	1082 1022	887	819	484
CEPN	3400	2937 2873	-	1666	1602	1602	1589	1440	1384	1384	1330	1273		1163	1080 1033	896	810	480

**Table 5:** Temperature ranges, percent mass loss, ratio between second and first mass losses and DTA peaks.

Sample	Process	TGA/DTG			DTA
		Temperature Range (°C)	Mass Loss (%)	Ratio *	Temperature Peak (°C)
NAP	1st step	139.4–365.3	97	-	283.8 (endo) 303.4 (exo)
	2nd step	365.3–491.0	1.50		430.3 (exo)
	Residue	491.0–1000.0	1.50		-
CTS	Dehydration	19.9–172.2	10.2	0.99	67.5 (endo)
	1st step	172.2–390.1	42.4		304.4 (exo)
	2nd step	390.1–609.9	42.0		544.2 (exo)
	Residue	609.9–1000.0	5.95		-
CN1	Dehydration	19.6–151.4	9.52	1.13	64.8 (endo)
	1st step	151.4–381.3	41.9		302.5 (exo)
	2nd step	381.3–617.3	47.3		522.0 (exo)
	Residue	617.3–1000.0	1.69		-
CEPN	Dehydration	23.6–171.5	9.45	1.17	56.4 (endo)
	1st step	171.5–399.0	38.7		301.2 (exo)
	2nd step	399.0–637.9	45.3		553.5 (exo)
	Residue	637.9–1000.0	6.60		-

* Ratio between values of percentage of mass losses observed in the second and first steps (2nd/1st).

**Table 6:** Values of the concentration of NAP in the equilibrium (partition constant), equilibrium time and released mass in solution in the experiments of pH 2.00 and 7.00.

Sample	pH	K_part_/mol L^−1^ *	m_NAP_ Dissolved/mg	Equilibrium Time/min
CN1	2.0	5.82 ± 0.03	0.67	17.54
	7.0	4.69 ± 0.02	0.54	25.48
CEPN	2.0	6.58 ± 0.07	0.76	104.3
	7.0	5.31 ± 0.03	0.61	36.68

* K_part_ = partition constant ± standard deviation, n = 3.

## Supplementary Materials

The following supporting information can be downloaded at: https://www.mdpi.com/article/10.3390/molecules27185801/s1, Figure S1: UV-vis diffuse reflectance spectroscopy spectra of NAP, CTS, CN1 (a) and CTS, CEPN (b) obtained in the diffuse reflectance mode; Figure S2: Diffractograms of NAP, CTS, CN1 and CEPN; Figure S3: DSC curves of NAP, CTS, CN1 and CEPN in N_2_ atmosphere flowing 50 mL min^−1^, sample mass of 4.0 ± 0.1 mg, in aluminum sample holder with a central pin hole in the lid and heating rate of 10 °C min^−1^; Figure S4: Chromatograms of CN1 (a) and CEPN (b) aliquots taken at different times at pH 2.00; Figure S5: Chromatograms of CN1 (a) and CEPN (b) aliquots taken at different times at pH 7.00 and Table S1: Parameters of the linear analytical curves obtained by HPLC for NAP at pH 2.00 and 7.00.

## Abbreviations

*CEPN*	Salt of naproxen and chitosan crosslinked with epichlorohydrin
*CN*	Salt of naproxen and chitosan
*COX*	Cyclo-oxygenase enzyme
*CT*	Chitin
*CTS*	Chitosan
*CTS–NAP*	Physical mixture of chitosan and naproxen
$\bar{DA}\;$	Degree of acetylation
$\bar{DD}\;$	Degree of deacetylation
$\bar{DS}$	Degree of substitution
*I_d_*	Crystallinity index
*K_part_*	Partition constant
$\bar{M}v$	Average viscometric molar mass
*NAP*	Naproxen
*[NAP]*	Equilibrium concentration of naproxen
*NSAID*	Non-steroidal anti-inflammatory drug
*PVP*	Polyvinylpyrrolidone
*S_d_*	Standard deviation
*t_R_*	PRetention time
