# Supplementary material for: Preparation, Characterization and Study of the Dissociation of Naproxen from Its Chitosan Salt
Source: Molecules. 2022 Sep 7;27(18):5801. doi: 10.3390/molecules27185801 (PMC9504068; doi:10.3390/molecules27185801)
Supplement: Supplementary file 1 [file molecules-27-05801-s001.zip › molecules-1896259-supplementary.pdf]

PREPARATION, CHARACTERIZATION AND STUDY OF THE DISSOCIATION OF NAPROXEN FROM ITS CHITOSAN SALTS

Ricardo S. Medeiros[1], Ana P.G. Ferreira[1], Tiago Venâncio[2], Éder T.G. Cavalheiro[1]*

Supplementary materials

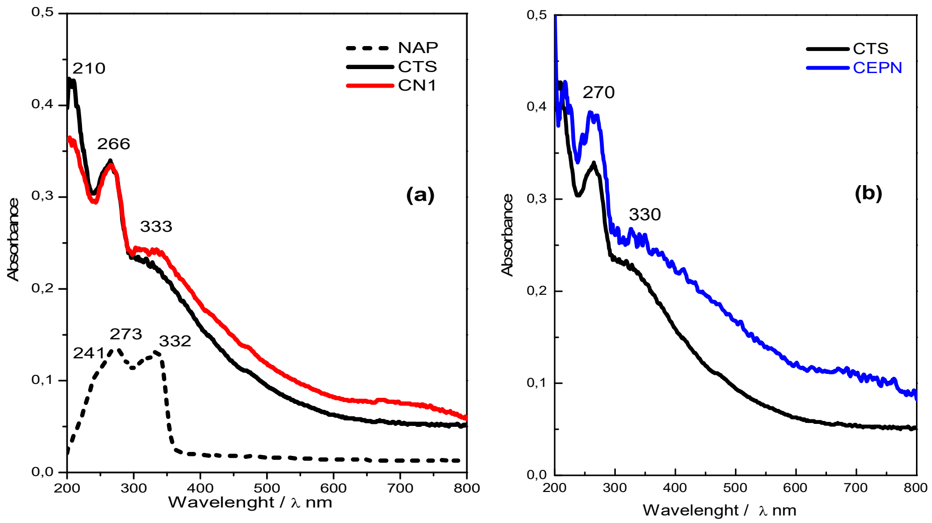

Figure S1. UV-vis diffuse reflectance spectroscopy spectra of NAP, CTS, CN1 (a) and CTS, CEPN (b) obtained in the diffuse reflectance mode.

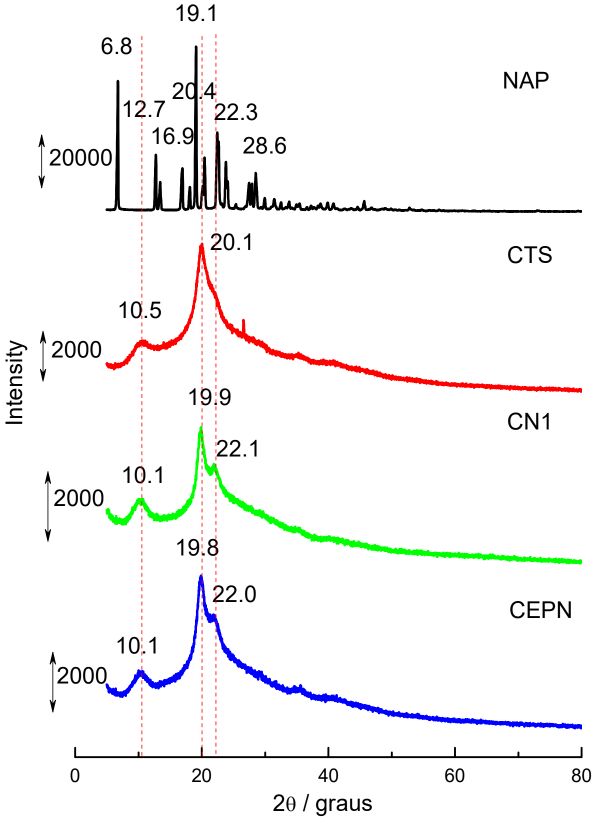

Figure S2. Diffractograms of NAP, CTS, CN1 and CEPN.

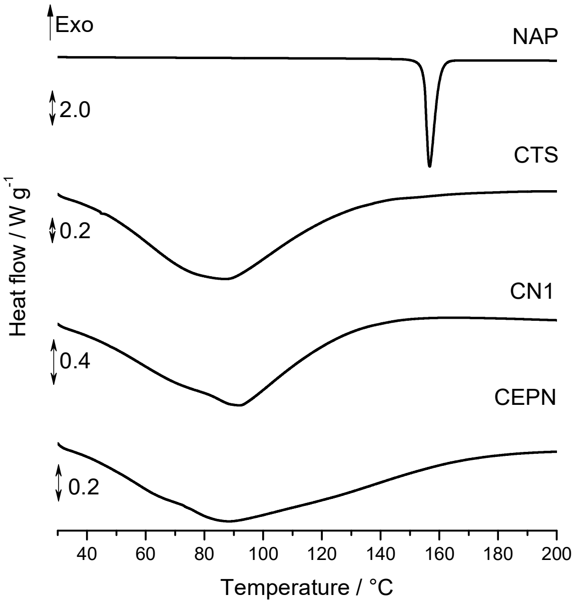

Figure S3. DSC curves of NAP, CTS, CN1 and CEPN in N_2 atmosphere flowing 50 mL min^{-1}, sample mass of 4.0 ± 0.1 mg, in aluminum sample holder with a central pin hole in the lid and heating rate of 10 °C min^{-1}.

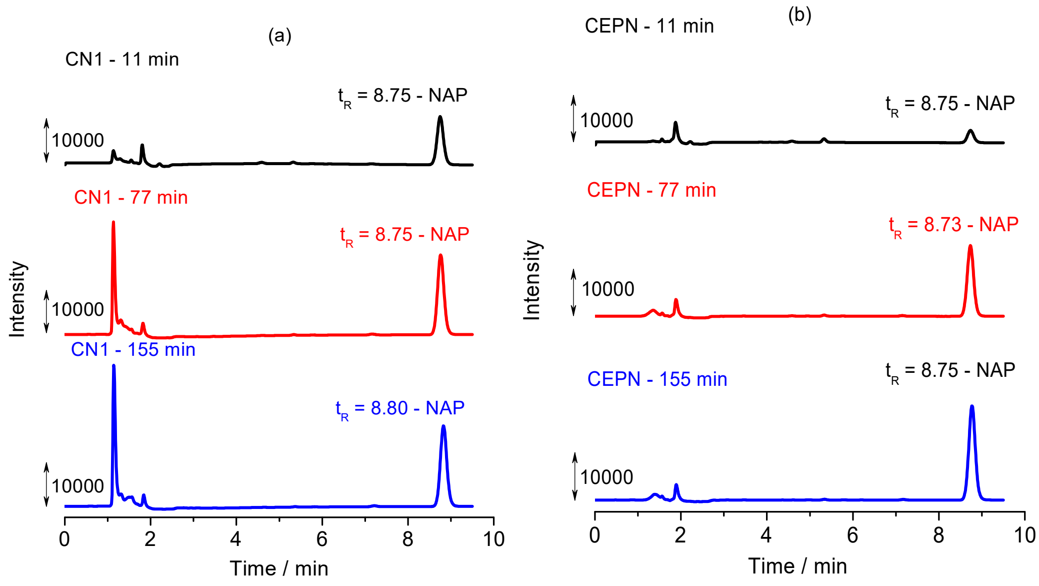

Figure S4. Chromatograms of CN1 (a) and CEPN (b) aliquots taken at different times at pH 2.00

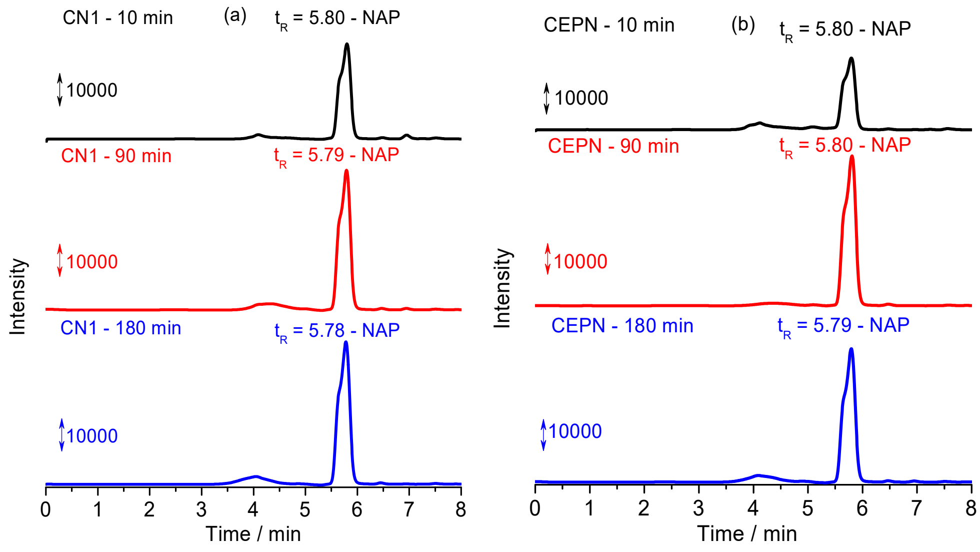

Figure S5. Chromatograms of CN1 (a) and CEPN (b) aliquots taken at different times at pH 7.00

Table S1. Parameters of the linear analytical curves obtained by HPLC for NAP at pH 2.00 and 7.00

**Medium**	**Intercept / 10^3**	**Sensitivity / 10^9 µmol L^{-1}**	**Linear region / µmol L^{-1}**	**Correlation coefficient , R^2**
**pH 2.00**	7.26	3.22	2.50-75.0	0.995
**pH 7.00**	25.5	12.8	1.00-125.0	0.998
